# 777. Implementation of Antimicrobial Impregnated Catheters to Reduce Central Line Associated Bloodstream Infections (CLABSI) in a Pediatric Setting

**DOI:** 10.1093/ofid/ofab466.974

**Published:** 2021-12-04

**Authors:** Ami B Patel, Sangeeta Schroeder, Armela Hadzic, Nadine A Schulz, Jannell A Bichl, Craig M Smith, Grant R Hahn, Erin DeRose, Catherine Collins, Jade Clark, Carolyn Wainer, Maria Hugo, Mary Lynn Rae, Michael A Evans, Eric L Vu, Lisa Sohn, Jerusha Pedersen, Anna M Lund, Angela Greenwood, Josephine A Davies, Antoinette Newburn, Shankar Rajeswaran, Ravi Jhaveri

**Affiliations:** 1 Ann & Robert H. Lurie Children's Hospital of Chicago, Chicago, Illinois; 2 Ann & Robert H Lurie Children's Hospital of Chicago, Chicago, Illinois; 3 Northwestern University Feinberg School of Medicine, Chicago, Illinois; 4 Lurie Children's Hospital, Chicago, Illinois; 5 Ann and Robert H. Lurie Children's Hospital of Chicago, Chicago, Illinois; 6 Lurie Childrens, Chicago, Illinois; 7 Ann & Rober H. Lurie Children's Hospital in Chicago, Chicago, Illinois; 8 Ann & Robert H. Lurie Children’s Hospital of Chicago, Chicago, Illinois; 9 Lurie Childrens Hospital, Chicago, Illinois; 10 Northwestern University/Lurie Children's Hospital of Chicago, Chicago, Illinois

## Abstract

**Background:**

Antimicrobial impregnated catheters (AIC) are one strategy to prevent CLABSI with existing data for central lines required for short duration, however, the strength of evidence, particularly for children, is lacking. Recent 3-year CLABSI data at our institution show 60 (51%) infections occurred in central lines within 8 weeks of insertion, suggesting an opportunity for evaluation of an intervention targeting this time frame. We implemented AIC to evaluate their effectiveness in reducing CLABSI standardized infection ratio (SIR) in patients requiring central venous access for less than 8 weeks. We also monitored for complications (malfunction, line exchange, fungal infection).

**Methods:**

A stepped wedge observational design was used to implement Minocycline + Rifampin impregnated catheters in a rolling fashion across the institution. Children > 3kg were eligible if admitted to a participating unit and required central venous access through a peripherally inserted central catheter (PICC), non-tunneled catheter, or tunneled non-cuffed femoral catheter for < 8 weeks. Units, prioritized based on CLABSI SIR, were added to the intervention monthly until AIC were used throughout the institution. A multidisciplinary team (infectious diseases and infection control experts, CLABSI leaders, unit-based physicians and nurses, proceduralists, supply chain) met weekly to facilitate implementation, assess for CLABSI and monitor for complications.

Figure 1. Study design.

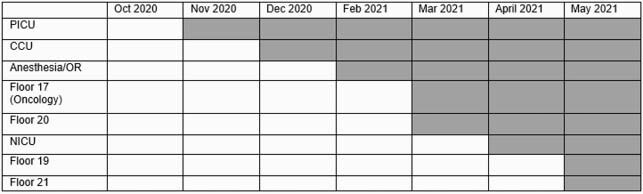

This figure describes the stepped wedge study design where units were phased into the invention on a rolling monthly basis allowing for comparison between and within units. The shaded boxes represent time periods when units were using antimicrobial impregnated catheters and the white boxes represent time periods when units were using standard non-impregnated catheters.

**Results:**

AIC were systematically implemented over a 7-month period. The institution’s CLABSI SIR decreased from 0.80 to 0.59 during this timeframe. There were no NHSH defined CLABSI in patients with an AIC during the intervention. Obstacles included shortage of catheters due to supply chain disruption, adjustment of technique for line insertion and cracked/broken lines. Infections and complications were reviewed by the multidisciplinary team and compared to historical rates with non-impregnated lines.

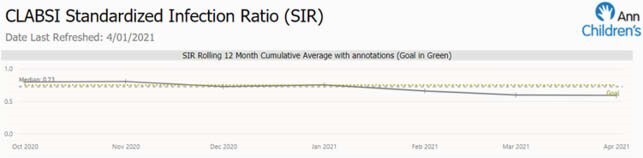

This figure shows the institution’s rolling 12-month SIR during the intervention period.

**Conclusion:**

CLABSI SIR decreased at our institution during the intervention period. While many efforts likely led to this reduction (optimizing maintenance bundle, unit based CLABSI initiatives), we believe the use of AIC contributed to this improvement. There were no pediatric-specific safety events identified during implementation.

**Disclosures:**

**Ravi Jhaveri, MD**, **AstraZeneca** (Consultant)**Dynavax** (Consultant)**Elsevier** (Other Financial or Material Support, Editorial stipend as Co-EiC, Clinical Therapeutics)**Hologic** (Consultant)**Seqirus** (Consultant)

